# Clarifying the Links between Perceived Stress and Depressiveness: a Longitudinal Study of COVID-19’s Effects on Adolescents in Germany

**DOI:** 10.1007/s10964-024-02012-8

**Published:** 2024-05-24

**Authors:** Gabriela Gniewosz

**Affiliations:** Department of Education, University Innsbruck, Innsbruck, Austria

**Keywords:** COVID-19, Depressiveness, Gender differences, Longitudinal research, Perceived stress

## Abstract

Young people are navigating an increasingly uncertain and unstable social and economic environment, further complicated by COVID-19. Individual resources and vulnerabilities, such as mental health and sensitivity to stress, play a significant role in how well youth adapt to the career paths and living conditions altered by the pandemic, a dynamic that is not yet well understood. This study examined the role of COVID-19 on the intertwined relation between perceived stress and depressiveness (negative and positive affect) in adolescents, focusing on gender differences. Longitudinal data from 673 German adolescents (M_age_ = 16.8 years, *SD*_age_ = 0.91; female = 59%) were collected in three waves, before (T1) and during the pandemic (T2, T3). Using Latent Change Score models, the bidirectional relation between perceived stress and depressiveness was analyzed, considering gender as a moderator. The results showed that adolescents who found their situation stressful were at risk of developing depressiveness at the outbreak of the pandemic and throughout its progression. As the pandemic progressed, an increase in positive affect was linked to heightened perceived stress. Gender-specific differences were particularly evident in the levels of perceived stress and depressiveness, with women being more vulnerable. This study highlights how vulnerabilities in stress perception affect adolescents’ mental health, with gender-specific differences underscoring the need for tailored mental health measures.

## Introduction

Since spring 2020, COVID-19 has significantly affected adolescents and young adults globally, especially concerning their mental health. Systematic reviews have shown a rise in mental health issues like depression, anxiety, and psychological distress among those ≤18 years, despite low physical symptom prevalence (Kauhanen et al., 2023; Orban et al., 2024). In Germany, the first lockdown from March to May 2020, involving school closures and strict social distancing, was followed by two pandemic waves with varying restrictions by the end of 2021 (see supplementary Figure S1). These changes led to increased mental health problems among young people in Germany (e.g., Mauz et al., 2023; Ravens-Sieberer et al., 2022). The ability of young people to navigate their developmental paths (e.g., school and after-school trajectories) through the pandemic may potentially be influenced by a range of factors, including pre-pandemic vulnerabilities. Notably, (pre-pandemic) mental health and sensitivity to stress are pivotal in determining how well adolescents adapt to these unprecedented challenges (e.g., Orban et al., 2024; Hawes et al., 2022). However, the interplay between mental health and perceived stress throughout the pandemic remains poorly understood, particularly when considering gender-specific differences. Male and female adolescents may differ significantly in their sensitivity to (unexpected) challenges and their subsequent adaptation strategies (see Rnic et al., 2023; Kozák et al., 2023). Building on this understanding, this study seeks to explore the bidirectional relation between adolescents’ depressiveness and perceived stress over a three-year period, encompassing both the pre-COVID-19 era and subsequent pandemic waves. This investigation specifically focuses on gender differences and aims to elucidate how these dynamics vary between young men and women.

### Mental Health during Adolescence and Young Adulthood

The transition from adolescence to young adulthood is a developmental phase associated with a heightened risk of developing deficits in mental health (Barkmann et al., 2016; Klasen et al., 2017). This life period can be demanding as young people seek autonomy, formulate their identity, and form stable relationships while facing puberty, school transitions, and post-school challenges (Branje et al., 2012). In terms of mental health, those who experience mental health problems such as depressiveness during the transition from adolescence to young adulthood are at a higher risk of developing mental health problems later in life (Fergusson et al., 2018; Johnson et al., 2018). However, although this transition is challenging for most young people, it affects adolescents’ mental health differently, suggesting individual differences in vulnerability. These variations arise from differences in available resources and preconditions (e.g., individual’s mental health status) which can make managing developmental tasks or environmental changes more challenging (e.g., Immel, Neumeier, & Peichl, 2022; Low & Mounts, 2022).

One aspect of mental health involves the subjective evaluation of one’s own emotional state (Keyes, 2006; Park et al. 2023). Positive affect reflects the presence of pleasurable emotions such as joy and enthusiasm, which are associated with better mental health (Keyes, 2006; Krohne et al., 2002), whereas negative affect involves experiencing distressing emotions like sadness and anger, often leading to dissatisfaction and impaired daily functioning (Immel et al., 2022; Casas & González-Carrasco, 2020). Literature places high levels of negative affect coupled with reduced positive affect (also known as anhedonia or loss of joy) within the spectrum of depression, as exemplified by the tripartite model of depression and anxiety (Clark & Watson, 1991). Depressiveness, as the dimensional equivalent of depression (Alt et al. 2021), is characterized by internalizing symptoms such as high negative and low positive affect without necessarily meeting the full clinical criteria for depression. Thus, depressiveness recognizes that depressive symptoms (i.e., individual’s emotional states) vary in intensity and duration among individuals beyond a clinical disorder.

Following this understanding, depressiveness is a valuable indicator for assessing adolescents’ ability to cope with developmental (i.e., educational transitions, see Young et al., 2019) or environmental challenges (i.e., COVID-19, see Alt et al., 2021). Generally, individual’s emotional state serves as a key mechanism for managing social interactions, where positive affect signals successful adaptation to the environment. In contrast, negative affect indicates struggles with social demands like interpersonal or pandemic-related stress (Spaderna et al., 2002). These emotional dynamics, particularly when increased negative affect and reduced positive affect prevails, can reflect how one copes with actual environmental challenges.

### The Role of Perceived Stress

Perceived stress is a psychological state reflecting individuals’ evaluation of life events as uncontrollable, unpredictable, and overwhelming. It describes individuals’ subjective need to constantly struggle with irritating problems and changes, as well as a lack of confidence in one’s own ability to deal with these difficulties (Phillips, 2013, p. 94). Perceived stress reflects the interaction between a person and their environment, which they experience as threatening, overwhelming, or consuming all their resources (Cohen et al., 1995; Lazarus & Folkman, 1984). Perceived stress refers to the need for an individual to adapt to certain life situations and can trigger the onset of mental health problems or worsen their course (Thoits, 2010). Based on this stress–vulnerability hypothesis, perceived stress may be detrimental to young people’s mental health, as it impairs the capacities necessary for successfully coping with developmental and pandemic-related challenges. This hypothesis suggests that perceived stress, especially in young people, can have a negative effect on mental health (Braet et al., 2022). The reason for this is twofold:

First, stress can impair the psychological and emotional capacities that are crucial for young people to successfully navigate the complex challenges of development, such as establishing their identity, building relationships, and making significant life choices (Rnic et al., 2023). These developmental challenges are inherently demanding, and the added pressure of stress can hinder a young person’s ability to manage them effectively (Fergusson et al., 2018; Johnson et al., 2018).

Second, when considering the unique context of the pandemic, young individuals face additional, unprecedented challenges. Measures to stop the spread of the virus, such as lockdowns and quarantines, led to reduced social contact, increased perceived threats, and the loss of daily routines and positive reinforcers. This situation compounds the usual stressors of youth, leading to an overload that can exceed their coping resources (Liu & Wang, 2021; Braet et al., 2022). This overload, occurring during a critical period of mental, emotional, and social development, may have heightened the risk of multiple stressors converging due to the co-occurrence of developmental challenges (Evans et al., 2013) and COVID-19 changes (Bonati et al., 2022; Kornilaki, 2022). Specifically, young people are potentially more prone to stress and negative developments, if two or more such risk factors co-occur. Thus, previous research has demonstrated that adolescents who reported higher levels of perceived stress due to COVID-19 restrictions tended to exhibit more pronounced depression symptoms, even those who did not experience mental health issues prior to the pandemic (Liu & Wang, 2021; Thorsen et al., 2022).

Yet, the stress–outcome relation may not always be a simple unidirectional effect. Rather, prior vulnerabilities like poor mental health may contribute to greater perceived stress. For instance, the stress–generation model suggests that individuals with depressive symptoms or certain maladaptive characteristics may generate stressful life events through their behavior, attitudes, or interpersonal interactions (Liu & Alloy, 2010; Santee et al., 2023). Individuals with depression symptoms often engage in behaviors or make decisions that provoke stressful life events, which, in turn, can intensify or prolong their feelings of depression. The stress–generation model is linked to the depression–distortion hypothesis (Richters, 1992), serving as an alternative explanation in the context of the relation between perceived stress and mental health. This model proposes that individuals with depressive symptoms are prone to disproportionately recall negative information, such as through overgeneralization or all-or-nothing thinking. Such cognitive biases negatively affects individual’s perspectives on social environments and, thus, reinforcing individual’s feelings of depressiveness. Both hypotheses suggest that depression negatively alters a person’s interaction with the world, impeding effective stress management as well as coping behavior (Arnaldo et al., 2022; Calvete et al., 2015) and, thus, contributing to an increased risk of becoming disproportionately stressed by the current situation. In the context of COVID-19, adolescents with higher levels of depressive symptoms before the pandemic may perceive the pandemic as more stressful and burdensome (depression–distortion hypothesis) and/or may be more inclined to behave in ways that reinforce their negative feelings (stress–generation model). Thus, pre-existing mental health problems may be a risk factor for negative developments during the pandemic (Kleine et al., 2023; van Loon et al., 2022).

### Gender Differences

Research, highlighting the explanatory role of gender, indicates that women are generally more susceptible to mental health issues like depressiveness and stress perception, particularly during COVID-19, showing greater levels of internalizing symptoms, feelings of depression and perceived stress than men (Hafstad et al., 2022; van Loon et al., 2022). Factors contributing to this include hormonal changes during puberty, societal pressures, and gender-specific life experiences (Rnic et al., 2023; Kozák et al., 2023). Women’s higher rates of depressiveness may also be influenced by their elevated susceptibility to stressors and emphasis on relationships due to gender role socialization (Giota & Gustafsson, 2017; Meiser & Esser, 2019). Additionally, women may be more likely to seek help and report their emotional distress, further contributing to the observed gender differences (Kuehner, 2017).

However, some recent studies challenge these findings, suggesting minimal or no gender differences in perceived stress and depressiveness, particularly when accounting for pre-existing vulnerabilities (Hawes et al., 2022; Rnic et al., 2023). For instance, COVID-19 studies have shown no significant gender differences in mental health outcomes (Orban et al., 2024; Hawes et al., 2022), with one study noting similar symptom changes across genders (van der Laan et al., 2022), and another indicating stronger symptom increases in men (Ludwig-Walz et al., 2022). This suggests that while gender differentiation in perceived stress and depressiveness is important, the differences may be smaller and less distinct than previously assumed.

## Current Study

**Fig. 1:**
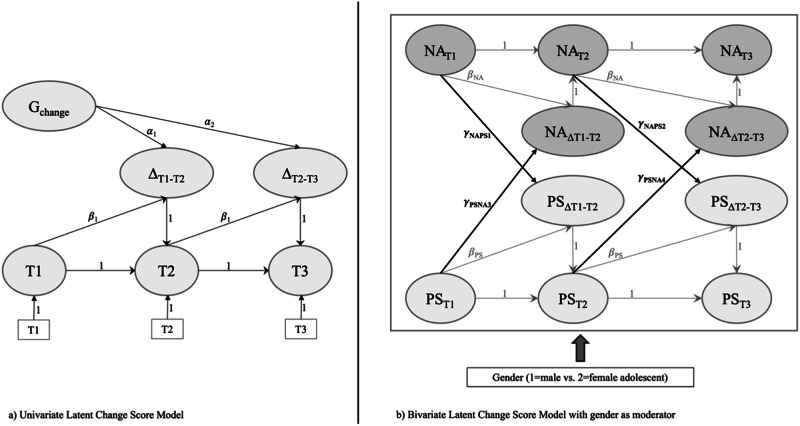
Schematic Overview. *Note*. Schematic overviewed of the specified LCSMs with univariate LCSM and bivariate LCSM with gender as a moderator variable (specified as a multi-group model); PS perceived stress; NA negative affect at three time points (T1–T3). G_change_ = General slope; Δ_T1–T2_/Δ_T2–T3_ = Proportional change in between two neighbored measurement points; Covariates and general slopes in the bivariate LCSM are not shown

Much existing research on the relationship between perceived stress and mental health in adolescence and young adulthood has focused primarily on the stress-to-outcome relation, often overlooking the interplay between these two aspects. Moreover, the exploration of this interplay between perceived stress and depressiveness from a gender perspective has been largely neglected. This oversight becomes particularly significant under the conditions of the pandemic, as it remains unclear how the relation between perceived stress and depressiveness varies by gender in such challenging times. The present study aimed to examine the moderating effect of gender on the association between adolescents’ perceived stress and depressiveness during COVID-19 (Fig. 1). This longitudinal study, capturing three waves of data collection during adolescents’ transition to early adulthood amidst COVID-19, investigates the bidirectional relation between perceived stress and feelings of depression (positive and negative affect). Given some indications of gender-based differences, the research examines the reciprocal associations between perceived stress and feelings of depression before (T1) and during the pandemic (T2, T3) from a gender perspective. According to the stress–vulnerability model, it is hypothesized that young people’s perceived stress would be positively linked with increased depressiveness (Hypothesis 1). Further, prior depressiveness in young people is expected to predict increased perceived stress during the pandemic, following the stress–generation model (Hypothesis 2). Based on the mixed results regarding gender-specific differences, the study also explores the moderating effect of adolescents’ gender (1 = male vs. 2 = female) on the (reciprocal) relationship between perceived stress and depressiveness during COVID-19 (Hypothesis 3).

## Methods

### Sample

The sample comprised 673 German adolescents, aged between 15 and 18 years (M = 16.82, *SD* = 0.91), with a minority (21.1%) being immigrants themselves or having at least one parent or grandparent who immigrated to Germany. Female adolescents were slightly overrepresented (female = 59%). Although compulsory schooling in Germany ends after 9 years, a significant majority of young people remain in secondary education (88.77%). This secondary education typically consists of three different forms: a higher track (“Gymnasium”; 61.8%), a middle track (“Realschule”; 21.2%), and a lower track (“Hauptschule”; 4.7%). At T3, ~40.7% of adolescents were still in school, with 16.5% having started vocational training and 16.2% having enrolled in a university program. For 55.8%, at least one parent in the family had a high school diploma or equivalent. Further, most adolescents lived with their parents before (98.8%) and during (97%) COVID-19. Some of the sample reported that they were poor, meaning that they and their families often had to forego something due to limited finances (T1: 6.5%; T3: 5.5%). At T2, 27.7% of the sample reported a decrease in their families’ household income due to the pandemic, and 24.5% stated that the first wave of the pandemic had significantly negatively affected them personally.

### Procedure

This study utilizes data from the comprehensive Panel Analysis of Intimate Relationships and Family Dynamics (pairfam) project in Germany (Huinink et al., 2011). The complex study design involved randomly selecting participants from private households across Germany, born in four different cohorts: 1991–1993, 1981–1983, 1971–1973, and the youngest, 2001–2003, added in 2019 as a new sample. The surveys have been conducted annually since 2008 and included an additional COVID-19 web survey during the first wave of the pandemic, between May 19 and July 13, 2020 (Brüderl et al., 2022; Walper et al., 2020). Thus, the first measurement point in 2019 (T1; January–April 2019) provided pre-pandemic information, while the COVID-19-web survey in 2020 (T2; May–July 2020) and a subsequent measurement in 2021 (T3; January–April 2021) captured data from the beginning and middle of the pandemic (see supplementary Figure S1). The T1 and T3 assessments were conducted using computer-assisted personal and self-administered questionnaire-based interviews in participants’ homes, carried out by trained interviewers from an independent institute (Brüderl et al., 2022). The data collection includes a series of standardized questionnaires on various topics (e.g., partnership, parenthood, social embeddedness; see, www.pairfam.de) and lasted about 60 min. Written parental consent was obtained for all participants under 18 years of age.

To gain insight into the stresses experienced during COVID-19, participants of the pairfam study were invited to partake in an extra web survey, which was carried out by an independent institute (Walper et al., 2020). Individuals were invited to complete an online questionnaire, which took about 15 min. In the pairfam COVID-19 survey, 9640 individuals were initially contacted, and out of these, 3154 respondents took part and gave valid information. Among them, 673 individuals were identified as being in middle adolescence (14–17 years old) and had participated in the study both before COVID-19 in 2019 (T1) and after the first wave of COVID-19 in 2021 (T3). These individuals were selected for further analysis.

### Measures

All variables were assessed from adolescents’ subjective perspectives. Supplementary Table S1 presents a detailed overview of the items.

#### Depressiveness

Depressiveness was measured at all measurement points using two trait scales from the State–Trait Depression Scale (STDS; Krohne et al., 2002). The first subscale represents negative affect, reflecting feelings such as sadness and depressiveness (e.g., “I feel sad”). The second subscale comprises positive affect, reflecting feelings such as happiness and trust (e.g., “I feel secure”). For each subscale, three items were used, rated on a Likert scale ranging from 1 (*almost never*) to 4 (*almost always*). The factor score reliability, as an indicator for internal consistency, was good for negative affect (*ω* [0.74, 0.82]) and positive affect (*ω* [0.73, 0.76]).

#### Perceived Stress

At all measurement points, individuals’ experienced stress or overload during the last four weeks (e.g., “How did you feel in the last four weeks? *Overburdened*”) was assessed with three items based on items from the German version of the Perceived Stress Questionnaire (Fliege et al., 2001). Adolescents responded on a scale ranging from 1 (*not at all*) to 5 (*absolutely*). Reliability was good at all measurement points (*ω* [0.86, 0.88]).

#### Covariates

To specify the main models as concisely as possible for the sample size, only a few important covariates were included. *Age*, which ranged from 15 to 18 years at T1, was included to control for age-specific developmental differences. *Families’ education* was included, representing the highest level (maximum) of parents’ education in years. Finally, information about *adolescents’ school track at T1* (coded as 1 = low to 2 = high) was also included.

#### The Moderator

Adolescents provided information about their gender (1 = male, 2 = female), a dichotomous variable that served as a moderator to test expected gender differences.

Descriptive statistics (M, *SD*), reliability estimates, and correlations for all indicators are shown in Table 1. Using a non-parametric MCAR test across time was non-significant (*p* = 0.113), indicating that there was no association between observed values and missingness (Jamshidian et al., 2014).

**Table 1:** Means, standard deviations, reliability, and bivariate correlations

	Variable		M/Freq.	SD/%	*ω*	1	2	3	4	5	6	7	8	9	10	11	12
1	Negative affect T1		1.74	0.57	0.74												
2	Negative affect T2		1.99	0.70	0.82	0.38**											
3	Negative affect T3		1.89	0.61	0.79	0.53**	0.43**										
4	Positive affect T1		3.14	0.60	0.75	−0.62**	−0.29**	−0.48**									
5	Positive affect T2		2.87	0.63	0.73	−0.29**	−0.60**	−0.35**	0.36**								
6	Positive affect T3		3.07	0.62	0.76	−0.40**	−0.32**	−0.64**	0.51**	0.48**							
7	Perceived stress T1		2.96	1.11	0.86	0.42**	0.29**	0.25**	−0.32**	−0.23**	−0.18**						
8	Perceived stress T2		2.73	1.13	0.87	0.23**	0.44**	0.32**	−0.20**	−0.38**	−0.25**	0.32**					
9	Perceived stress T3		3.15	1.10	0.88	0.26**	0.31**	0.44**	−0.21**	−0.26**	−0.35**	0.36**	0.42**				
10	Gender T1	m	276	41.01%	—	0.12**	0.25**	0.16**	−0.08*	−0.19**	−0.11*	0.21**	0.25**	0.21**			
		f	397	58.99%													
11	Age T1		16.82	0.91	—	−0.02	−0.01	−0.09*	0.03	−0.02	0.06	0.01	0.00	−0.06	−0.01		
12	Highest school track T1	l	179	26.60%	—	−0.01	−0.00	0.03	0.07	0.04	−0.01	0.06	0.10*	0.08	0.02	0.24**	
		h	416	61.81%													
16	Education of family T1		5.78	1.66	—	−0.00	−0.02	0.02	0.05	0.01	−0.01	0.10*	0.01	0.03	−0.07	0.02	0.38**

*N* = 673; M/Freq and SD/% are used to represent mean/frequencies for categorical data and standard deviation/percentage for categorical data, respectively. *ω* represents the factor score reliability. All variables are reported by adolescents. T1 = measurement point before COVID-19; T2 and T3 = measurement points during COVID-19; Gender: f female (*n* = 397) and m male (*n* = 276). School track: l = not highest secondary school track (Realschule or Hauptschule) and h = highest secondary school track (Gymnasium). Education of family: Highest level (maximum) of family’s education in years

*p < 0.05; **p < 0.01

### Analytic Plan

Data and syntaxes that support the findings of this study are openly available in the Open Science Framework (https://osf.io/mj924/). All analyses were conducted in R version 4.0.2 (RCoreTeam, 2023), with the R package lavaan (Rosseel, 2012), using a robust maximum likelihood (ML) estimator (Finney & DiStefano, 2006). The full information ML (FIML) adjustment method was applied to account for missing data. In addition to the *χ*^2^ test statistic, several fit indices were utilized to evaluate goodness of fit (Hu & Bentler, 1999), including the Root-Mean-Square Error of Approximation (RMSEA ≤ 0.05), Standardized Root Mean-Square Residual (SRMR ≤ 0.08), Comparative Fit Index (CFI) and Tucker-Lewis Index (TLI ≥ 0.90).

Preliminarily, invariance over time and gender were tested for positive and negative affect and perceived stress. For each construct, the configural invariance (i.e., no parameter restrictions) model was compared with the weak invariance model (equal factor loadings across time/gender), strong invariance model (equal item intercepts across time/gender), and strict invariance model (equal residual variances across time/gender). Since chi-square difference tests are affected by complexity of the model and sample size (Cheung & Rensvold, 2002), the model comparisons were conducted considering changes in fit indices (Chen, 2007). Thus, the ΔCFI (CFI change ≤−0.010), the ΔRMSEA (RMSEA change ≤0.015) and the ΔSRMR criterion (SRMR change ≤ 0.030) were used (Chen, 2007). The findings indicated strict measurement invariance for all constructs, both longitudinally (see supplementary Tables S2a–S2c) and across genders (see supplementary Tables S3a–S3c). Therefore, changes over time and differences between males and females can be meaningfully interpreted as they are not due to alterations in the measurement models. Based on the invariant models, composite scores for perceived stress and negative and positive affect were used for the proceeding models.

Due to space restrictions, the next steps of the analysis will be described briefly (see Fig. 1). An overview of the specified models, including a more detailed explanation of the specifications, is depicted in supplementary Table S4.

In step two, time-invariant univariate latent change structural models (LCSMs; Kievit et al., 2018; McArdle, 2009) were specified to estimate how one construct changed over time, providing information about constant and proportional change and the autoregressive effects of the change scores (McHugh Power et al., 2019). While constant change represented overall changes between T1 and T3, proportional change reflected local changes between the two neighbored measurement points (T1→T2; T2→T3). The autoregressions of the change scores described whether any given change score was determined by the previous change score (Kievit et al., 2018; McHugh Power et al., 2019). In total, three univariate LCSMs were specified to estimate “pure” changes in perceived stress (BM_PS_1), positive affect (BM_PA_2), and negative affect (BM_NA_3).

Based on the univariate LCSMs, two separate bivariate LCSMs (Kievit et al., 2018; McArdle, 2009) were specified to understand how perceived stress related to negative (M_PSNA_1) and positive (M_PSPA_2) affect over time. Specifying negative and positive affect in two separate (and equally specified) models seemed conducive to gaining a deeper understanding of the specific coupled relationships between perceived stress and the two constructs of depressiveness over time. The bivariate LCSM approach allowed for the simultaneous modeling of changes in two constructs over time, distinguishing between constant and proportional effects (Kievit et al., 2018; McHugh Power et al., 2019). Coupling parameters were additionally introduced to examine the time-dependent effects of the previous levels of one construct on local changes in the second construct and vice versa, accounting for the interplay between these two variables over time. Finally, three exogenous variables were added to these two bivariate LCSMs to test the predictive value of age, families’ education, and school track on the proportional changes.

In step three, gender was included in the two bivariate LCSMs as a moderator. Specifically, allowing for the simultaneous modeling and testing of the relationships between perceived stress and negative affect (MG_PSNA_1) and between perceived stress and positive affect (MG_PSPA_2) across the gender groups. Each model was specified as invariant across time and gender. Gender-specific differences were tested for using the Wald test (Bollen, 1989).

## Results

### Pre-analyses

Time-invariant univariate LCSMs without prediction between latent variables (i.e., without coupling parameters) and exogenous variables (i.e., covariates) were tested as “baseline” models to estimate latent means for and changes in negative and positive affect and perceived stress. An acceptable to good fit to the data (see supplementary Table S4, last column) was shown for the baseline models of perceived stress (BM_PS_1: *χ*^*2*^(2) = 1.740, *p* = 0.410, RMSEA = 0.010, CFI = 0.998, TLI = 0.990), negative affect (BM_NA_2: *χ*^2^(2) = 4.03, *p* = 0.133, RMSEA = 0.039, CFI = 0.993, TLI = 0.980) and positive affect (BM_PA_3*:χ*^*2*^(2) = 4.17, *p* = 0.133, RMSEA = 0.040, CFI = 0.993, TLI = 0.979). Regarding general changes over time in perceived stress (MSPs = 2.52, *p* < 0.001), negative affect (MSNa = 2.34, *p* < 0.001), and positive affect (MSPa = 3.54, *p* < 0.001), participants reported an increase between T1 and T3 (see Table 2, upper half).

**Table 2:** Latent means and changes between T1 and T3 (overall sample and gender-specific subsamples)

	Negative Affect						Positive Affect						Perceived Stress					
	M	SE	*p*	S^2^	SE	*p*	M	SE	*p*	S^2^	SE	*p*	M	SE	*p*	S^2^	SE	*p*
Overall Sample																		
Intercept (level)	1.74	0.02	<0.001	0.32	0.02	<0.001	3.14	0.02	<0.001	0.37	0.02	<0.001	2.96	0.04	<0.001	1.22	0.05	<0.001
General Change (T1–T3)	2.34	0.15	<0.001	0.33	0.06	<0.001	3.54	0.24	<0.001	0.30	0.06	<0.001	2.62	0.23	<0.001	0.25	0.15	0.083
Gender Difference Intercept (Level)																		
Male	1.66	0.03	<0.001	0.29	0.03	<0.001	3.20	0.03	<0.001	0.31	0.03	<0.001	2.68	0.06	<0.001	1.10	0.07	<0.001
Female	1.79	0.03	<0.001	0.34	0.03	<0.001	3.10	0.03	<0.001	0.40	0.03	<0.001	3.15	0.06	<0.001	1.22	0.06	<0.001
W (*df*), *p*	9.71(1)		0.002	1.09(1)		0.296	5.12(1)		0.024	5.39(1)		0.020	30.89(1)		<0.001	1.44(1)		0.229
Gender Difference General Changes (T1–T3)																		
Male	2.21	0.13	<0.001	0.29	0.06	<0.001	3.85	0.24	<0.001	0.33	0.07	<0.001	2.44	0.19	<0.001	0.28	0.13	0.032
Female	2.55	0.15	<0.001	0.36	0.06	<0.001	3.61	0.22	<0.001	0.31	0.06	<0.001	2.93	0.24	<0.001	0.26	0.15	0.078
W (*df*), *p*	36.11(1)		<0.001	3.03(1)		0.082	20.45(1)		<0.001	0.09(1)		0.763	38.34(1)		<0.001	0.066(1)		0.798

*N* = 673; Male: *n* = 276/Female: *n* = 397. Estimated latent means and standard errors (SE) are shown. Information is based on baseline univariate LCSMs (no predictions). The robust ML estimator was used, with FIML for handling missing data. Model fit for the univariate LCSM for negative affect: *χ*^*2*^(2) = 4.03, *p* = 0.133, RMSEA = 0.039, CFI/TLI = 0.993/0.980. Model fit for the univariate LCSM for positive affect: *χ*^*2*^(2) = 4.17, *p* = 0.133, RMSEA = 0.040, CFI/TLI = 0.993/0.979. Model fit for the univariate LCSM for perceived stress: *χ*^*2*^(2) = 1.740, *p* = 0.410, RMSEA = 0.010, CFI/TLI = 0.998/0.990. Model fit for the univariate multi-group LCSM for negative affect: *χ*^*2*^(6) 9.763, *p* = 0.135, RMSEA = 0.043, CFI/ TLI = 0.987/ 0.973. Model fit for the univariate multi-group LCSM for positive affect: *χ*^*2*^(6) 15.64, *p* = 0.016, RMSEA = 0.069, CFI/TLI = 0.968/0.935. Model fit for the univariate multi-group LCSM for perceived stress: *χ*^*2*^(6) 4.42, *p* = 0.621, RMSEA = 0.010, CFI/TLI = 0.998/0.995

Additionally, three univariate multigroup LCSMs showing an acceptable model fit were specified to estimate group-specific differences in means and changes (MG_univariate_BM_PS_1: *χ*^*2*^(*6*) 4.42, *p* = 0.621, RMSEA = 0.010, CFI = 0.998, TLI = 0.995; MG_univariate_BM_NA_2: *χ*^*2*^(*6*) 9.763, *p* = 0.135, RMSEA = 0.043, CFI = 0.987, TLI = 0.973; MG_univariate_BM_PA_3: *χ*^*2*^(*6*) 15.64, *p* = 0.016, RMSEA = 0.069, CFI = 0.968, TLI = 0.935; see supplementary Table S4, last column). Female adolescents reported higher levels of perceived stress (W*(1)* = 30.89, *p* < 0.001) and negative affect (W*(1)* = 9.71, *p* = 0.002) but lower levels of positive affect (W*(1)* = 5.39, *p* = 0.020) than male adolescents. Further, women reported a greater average increase in perceived stress (W*(1)* = 38.34, *p* < 0.001) and negative affect (W*(1)* = 36.11, *p* < 0.001) but a smaller increase in positive affect (W*(1)* = 20.45, *p* < 0.001) between T1 and T3 than men (see Table 2, lower half).

### Main Analyses

The extended LCSMs, including bidirectional paths and covariates, showed an acceptable to good fit to the data (see supplementary Table S4, last column) for the model of perceived stress and negative affect (M_PSNA_1: *χ*^*2*^ (*7*) = 8.754, *p* = 0.272, RMSEA = 0.019, SRMR = 0.016, CFI = 0.998, TLI = 0.990) as well as for perceived stress and positive affect (M_PSPA_2: *χ*^*2*^ (*7*) = 21.776, *p* = 0.059, RMSEA = 0.026, SRMR = 0.016, CFI = 0.988, TLI = 0.964). All information about the estimated parameters in the models is reported in Tables 3 and 4.

**Table 3:** Perceived stress and negative affect (overall sample and gender-specific subsamples)

	Regression Slope										ΔDifference	
	Overall Sample				Men			Women			Men vs. Women	
	*B*	SE	*p*	95% CI	*B*	SE	*p*	*B*	SE	*p*	W (*df*)	*p*
Changes in Perceived Stress from T1 to T2												
Perceived stress T1 (feedback parameter)	**−1.02**	**0.07**	**<0.001**	[−1.15; −0.89]	**−1.02**	**0.10**	**<0.001**	**−1.01**	**0.08**	**<0.001**	0.017 (*1*)	0.895
Negative affect T1	0.03	0.11	0.778	[−0.18; 0.25]	0.28	0.21	0.188	−0.17	0.14	0.203	1.323 (*1*)	0.250
Age	0.01	0.04	0.904	[−0.08; 0.09]	−0.04	0.06	0.511	0.03	0.06	0.551	0.182 (*1*)	0.669
Education of family	−0.01	0.04	0.784	[−0.09; 0.07]	0.04	0.06	0.563	−0.01	0.05	0.817	0.348 (*1*)	0.555
Education of adolescent (low vs. high track)	−0.01	0.04	0.799	[−0.09; 0.07]	0.07	0.06	0.227	−0.05	0.05	0.309	2.494 (*1*)	0.114
Changes in Perceived Stress from T2 to T3												
Perceived stress T2 (feedback parameter)	**−1.02**	**0.07**	**<0.001**	[−1.15; −0.89]	**−1.02**	**0.10**	**<0.000**	**−1.01**	**0.08**	**<0.001**	0.017 (*1*)	0.895
Negative affect T2	0.15	0.11	0.185	[−0.07; 0.36]	**0.53**	**0.23**	**0.019**	−0.10	0.13	0.436	2.934 (*1*)	0.087
Age	**−1.00**	**0.04**	**0.019**	[−0.18; −0.02]	−0.07	0.06	0.232	−0.11	0.06	0.070	0.182 (*1*)	0.669
Education of family	−0.01	0.04	0.813	[−0.10; 0.08]	0.03	0.06	0.670	−0.01	0.06	0.823	0.212 (*1*)	0.646
Education of adolescent (low vs. high track)	**0.13**	**0.04**	**0.004**	[0.04; 0.21]	0.06	0.07	0.450	**0.17**	**0.06**	**0.003**	1.636 (*1*)	0.201
Changes in Negative Affect from T1 to T2												
Negative affect T1 (feedback parameter)	**−1.22**	**0.09**	**<0.001**	[−1.40; −1.05]	**−1.12**	**0.17**	**<0.001**	**−1.31**	**0.10**	**<0.001**	0.959 (*1*)	0.327
Perceived stress T1	**0.09**	**0.03**	**0.007**	[0.02; 0.15]	**0.13**	**0.06**	**0.021**	0.05	0.04	0.156	3.232 (*1*)	0.072
Age	−0.01	0.03	0.993	[−0.05; 0.05]	−0.02	0.04	0.605	0.01	0.03	0.772	0.346 (*1*)	0.556
Education of family	−0.02	0.03	0.401	[−0.07; 0.03]	0.03	0.04	0.475	−0.03	0.03	0.453	1.067 (*1*)	0.302
Education of adolescent (low vs. high track)	−0.01	0.03	0.830	[−0.05; 0.04]	0.04	0.04	0.271	−0.03	0.03	0.319	2.193 (*1*)	0.139
Changes in Negative Affect from T2 to T3												
Negative affect T2 (feedback parameter)	**−1.22**	**0.09**	**<0.001**	[−1.40; −1.05]	**−1.12**	**0.17**	**<0.001**	**−1.31**	**0.10**	**<0.001**	0.959 (*1*)	0.327
Perceived stress T2	**0.09**	**0.04**	**0.023**	[0.01; 0.17]	**0.20**	**0.08**	**0.011**	0.04	0.05	0.379	**5.839 (*1*)**	**0.016**
Age	−0.04	0.02	0.075	[−0.08; 0.01]	−0.04	0.03	0.263	−0.04	0.03	0.268	0.001 (*1*)	0.977
Education of family	0.01	0.02	0.751	[−0.04; 0.06]	−0.01	0.03	0.855	0.03	0.04	0.485	0.409 (*1*)	0.522
Education of adolescent (low vs. high track)	−0.04	0.02	0.092	[−0.09; 0.01]	−0.04	0.03	0.192	−0.04	0.04	0.239	0.002 (*1*)	0.965

*N* = 673; Male: *n* = 276/Female: *n* = 397. Unstandardized regression coefficient (*B*), standard errors (SE), and *p*-values are shown. Robust ML estimator was used, with FIML for handling missing data. 95% CI = 95% confidence interval; T1 = measurement point before COVID-19; T2 and T3 = measurement points during COVID-19. Model fit for bivariate LCSM: *χ*^*2*^(*7*) = 8.754, *p* = 0.272, RMSEA/SRMR = 0.019/0.016, CFI/TLI = 0.998/0.990. Model fit for full multigroup bivariate LCSM: *χ*^*2*^(*14*) = 22.87, *p* = 0.021, RMSEA/SRMR = 0.043/0.027, CFI/TLI = 0.988/0.945. Values in bold represent significant effects

**Table 4:** Perceived Stress and Positive Affect (Overall Sample and Gender-Specific Subsamples)

	Regression Slope							ΔDifference				
	Overall Sample				Men			Women			Men vs. Women	
	*B*	SE	*p*	95% CI	*B*	SE	*p*	*B*	SE	*p*	W (*df)*	*p*
Changes in Perceived Stress from T1 to T2												
Perceived stress T1 (feedback parameter)	**−1.07**	**0.06**	**<0.001**	[−1.19;−0.95]	**−1.12**	**0.08**	**<0.001**	**−1.03**	**0.09**	**<0.001**	0.620 (1)	0.431
Positive affect T1	0.19	0.12	0.116	[−0.05; 0.43]	0.27	0.19	0.147	0.15	0.15	0.316	0.279 (1)	0.597
Age	−0.01	0.04	0.741	[−0.10; 0.07]	−0.02	0.06	0.800	−0.01	0.06	0.989	0.032 (1)	0.858
Education of family	−0.04	0.04	0.370	[−0.13; 0.05]	0.03	0.07	0.645	−0.05	0.06	0.341	0.936 (1)	0.333
Education of adolescent (low vs. high track)	**0.18**	**0.09**	**0.038**	[0.01; 0.35]	0.09	0.13	0.471	**0.23**	**0.12**	**0.039**	0.554 (1)	0.457
Changes in Perceived Stress from T2 to T3												
Perceived stress T2 (feedback parameter)	**−1.07**	**0.06**	**<0.001**	[−1.19; −0.95]	**−1.12**	**0.08**	**<0.001**	**−1.03**	**0.09**	**<0.001**	0.620 (1)	0.431
Positive affect T2	**0.39**	**0.14**	**0.006**	[0.12; 0.67]	**0.45**	**0.21**	**0.034**	0.36	0.19	0.053	0.241 (1)	0.624
Age	−0.**10**	**0.05**	**0.04**	[−0.10; 0.07]	−0.08	0.07	0.266	−0.10	0.06	0.116	0.061 (1)	0.806
Education of family	−0.01	0.05	0.902	[−0.13; 0.05]	0.07	0.07	0.295	−0.01	0.06	0.853	0.822 (1)	0.365
Education of adolescent (low vs. high track)	**0.13**	**0.04**	**0.002**	[0.01; 0.35]	0.07	0.06	0.277	**0.18**	**0.06**	**0.001**	1.813 (1)	0.178
Changes in Positive Affect from T1 to T2												
Positive affect T1 (feedback parameter)	**−1.31**	**0.06**	**<0.001**	[−1.44; −1.19]	**−1.15**	**0.14**	**<0.001**	**−1.37**	**0.07**	**<0.001**	1.993 (1)	0.158
Perceived stress T1	−0.02	0.03	0.463	[−0.07; 0.03]	−0.04	0.04	0.355	−0.01	0.03	0.738	0.264 (1)	0.607
Age	−0.02	0.02	0.320	[−0.07; 0.02]	0.03	0.04	0.390	**−0.07**	**0.03**	**0.024**	4.519 (1)	0.033
Education of family	−0.01	0.02	0.641	[−0.06; 0.04]	−0.05	0.04	0.137	−0.01	0.03	0.953	1.18 (1)	0.278
Education of adolescent (low vs. high track)	**0.08**	**0.04**	**0.046**	[0.01; 0.16]	0.08	0.05	0.141	0.08	0.06	0.169	0.01 (1)	0.984
Changes in Positive Affect from T2 to T3												
Positive affect T2 (feedback parameter)	**−1.31**	**0.06**	**<0.001**	[−1.44; −1.19]	**−1.15**	**0.14**	**<0.001**	**−1.37**	**0.07**	**<0.001**	1.993 (1)	0.158
Perceived stress T2	0.07	0.04	0.072	[−0.01; 0.15]	0.10	0.06	0.101	0.06	0.05	0.248	0.10 (1)	0.756
Age	0.02	0.03	0.378	[−0.03; 0.08]	0.07	0.04	0.063	−0.02	0.04	0.678	2.711 (1)	0.099
Education of family	−0.02	0.03	0.452	[−0.08; 0.03]	−0.01	0.04	0.935	−0.04	0.04	0.294	0.388 (1)	0.533
Education of adolescents (low vs. high track)	0.01	0.02	0.933	[−0.04; 0.05]	−0.06	0.03	0.059	0.04	0.03	0.236	4.834 (1)	0.027

*N* = 673; Male: *n* = 276/Female: *n* = 397. Unstandardized regression coefficient (*B*), standard errors (SE), and *p-*values are shown. Robust ML estimator was used, with FIML for handling missing data. 95% CI = 95% confidence interval; T1 = measurement point before COVID-19; T2 and T3 = measurement points during COVID-19. Model fit for full bivariate LCSM: *χ*^*2*^(*7*) = 21.776, *p* = 0.059, RMSEA/SRMR = 0.026/0.016, CFI/TLI = 0.988/0.964. Model fit for full multigroup bivariate LCSM: *χ*^*2*^(*14*) = 39.47, *p* = 0.021, RMSEA/SRMR = 0.039/0.034, CFI/TLI = 0.980/0.941. Values in bold represent significant effects

The effects of control variables were negligible overall. Regarding the first LCSM (M_PSNA_1), attending a higher school track at T1 corresponded with a smaller increase in perceived stress (*B* = 0.13, *p* = 0.004). Additionally, older adolescents at T1 reported a smaller increase in perceived stress (*B* = −1.00, *p* = 0.019). Regarding the second LCSM (M_PSPA_2), being on a higher school track was associated with a greater increase in perceived stress between T1 and T2 (*B* = 0.18, *p* = 0.038) and between T2 and T3 (*B* = 0.13, *p* = 0.002) and a greater increase in positive affect between T1 and T2 (*B* = 0.08, *p* = 0.046). Further, older adolescents showed a smaller increase in perceived stress (*B* = −0.10, *p* = 0.040).

Concerning the relationship between perceived stress and negative affect (M_PSNA_1), only the coupling parameter from perceived stress at T1 to proportional changes in negative affect was significant. Higher pre-pandemic stress was linked with greater increases in negative affect between T1 and T2 (*B* = 0.09, *p* = 0.007) and between T2 and T3 (*B* = 0.09, *p* = 0.023). There were no further predictive relationships between negative affect and perceived stress. The feedback parameters indicated that adolescents who had high scores for perceived stress and negative affect at the previous time point reported a smaller increase at the next time point (for perceived stress: *B* = −1.02, *p* < 0.001; for negative affect: *B* = −1.22, *p* < 0.001).

In terms of how perceived stress and positive affect were linked (M_PSPA_2), higher values in positive affect at T2 were associated with greater increases in perceived stress between T2 and T3 (*B* = 0.39, *p* = 0.006). However, adolescents who were highly positive during the initial phase of the pandemic (T2) reported a greater increase in stress after the first acute phase of the pandemic. There were no further predictive relationships between perceived stress and positive affect. The feedback parameters indicated that adolescents who had high scores for perceived stress and positive affect at the previous time point reported a smaller increase at the next time point (for perceived stress: *B* = −1.07, *p* < 0.001; for positive affect: *B* = -1.31, *p* < 0.001).

Regarding the role of gender, all bivariate LCSMs were extended by the moderator *gender* (MG_PSNA_1, MG_PSPA_1). All models demonstrated an acceptable fit (see supplementary Table S4, last column) for the model of perceived stress and negative affect (MG_PSNA_1: *χ*^*2*^ (*14*) = 22.87, *p* = 0.021, RMSEA = 0.043, SRMR = 0.027, CFI = 0.988, TLI = 0.945) as well as for the model of perceived stress and positive affect (MG_PSPA_2: *χ*^*2*^ (*14*) = 39.47, *p* = 0.021, RMSEA = 0.039, SRMR = 0.034, CFI = 0.980, TLI = 0.941). However, the moderating effect of gender on the relationship between perceived stress and the two facets of depressiveness yielded only slight differences across gender groups (see Table 3, last two columns): Higher perceived stress at T2 was associated with greater increases in negative affect between T2 and T3 (*B* = 0.20, *p* = 0.011) for men but not for woman (W*(1)* = 5.84, *p* = 0.016). No further significant differences were found between the gender groups.

## Discussion

Prior research primarily focused on how perceived stress affects mental health outcomes during adolescence and young adulthood, often overlooking the bidirectional relation between these factors. The role of gender in these dynamics has also been inadequately explored, particularly during the pandemic when the nature of these relations may differ significantly between male and female adolescents. To address this gap, this study investigated the bidirectionality between perceived stress and depressiveness throughout the pandemic. By extending previous research, our findings aimed to elucidate the complex ways in which perceived stress and depressiveness interrelate under pandemic pressures, with a focus on gender-specific differences.

### General Trends in Perceived Stress and Depressiveness

A significant increase in perceived stress and negative affect was observed, with a simultaneous increase in positive affect, from the initial (T1) to the third (T3) assessment. The findings for negative affect and perceived stress support the broader pattern witnessed during the pandemic, which signified a heightened prevalence of negative emotions and elevated stress levels among adolescents (Houghton et al., 2016; Kleine et al., 2023). Factors such as the loss of peer interactions, continued social isolation, limited leisure activities and uncertainties about upcoming post-school pathways were some of the challenges adolescents experienced due to the pandemic. A year after the pandemic’s initial wave in 2021, mental health levels in young people had not returned to pre-pandemic standards, indicating lasting negative effects. This persisted despite relaxed pandemic measures, with increased mental burdens like uncertainty about vaccines exacerbating depressiveness and stress (Ravens-Sieberer et al., 2022; van der Laan et al., 2022).

Extending prior findings, there was a significant increase in positive affect over the observed period, showing that during the pandemic, both negative and positive affective developments were possible. This supports previous research, showing that positive and negative affect can occur simultaneously in young adults (Diener & Emmons, 1984). Thus, the level of positive affect a person experiences is not necessarily related to the level of negative affect they experience and vice versa (Gill et al., 2017). For instance, despite the pandemic situation is generally experienced as restrictive and threatening, some adolescents also reported positive evaluations of the pandemic related to self-care and reflection (Bell et al., 2023). Thus, this study points to the need to differentiate forms of depressiveness.

However, the findings also demonstrated significant interindividual variations in changes in perceived stress and negative and positive affect over the study period (T1–T3), indicating that young people coped with lockdowns in diverse ways. Notably, despite limited in-person contact, some adolescents seemed to exhibit greater resilience and employed more effective coping strategies such as active coping strategies (seeking support, cognitive restructuring) during the pandemic (Budimir et al., 2021; Foster et al. 2023).

### Relationships between Perceived Stress and Depressiveness Over Time

The link between perceived stress and negative affect in adolescents is well-established. Confirming Hypothesis 1, the results demonstrate that pre-pandemic perceived stress was associated with an increase in negative affect during both the early (T1–T2) and later (T2–T3) phases of the pandemic. In terms of negative affect, these findings support the hypothesis that pre-pandemic stress heightened the likelihood of experiencing adverse mental health outcomes during the pandemic (Liu & Wang, 2021; Thorsen et al., 2022). It is plausible that adverse pre-pandemic perceived stress increased susceptibility to psychological distress during challenging times, thereby amplifying the potential for negative effects. Drawing from the stress–vulnerability model, how adolescents handled stress before the pandemic was closely interconnected with their responses to the unforeseen events of the pandemic (Liu & Alloy, 2010; Thoits, 2010). Therefore, adolescents who generally evaluated their abilities to and competence in coping with (unexpected) situations as insufficient were particularly vulnerable to negative affective developments during the pandemic.

It is important to mention that the lack of a significant “reversed” link between depressiveness—marked by low positive affect and high negative affect—and an increase in perceived stress, as suggested by Hypothesis 2, could be due to several reasons. One reason might be that the negative effects of depressiveness mainly show when depressive symptoms are extremely severe. However, the sample utilized in this study largely falls within the non-clinical spectrum of negative and positive affect, which may account for the findings.

Interestingly, the analysis of this “reversed” relation, yielded an unexpected result, underscoring the intricate impact of the pandemic on the lives and development of young individuals. For T2, it was observed that higher levels (and not reduced levels) of positive affect during the aftermath of the acute pandemic phase were associated with an increase in perceived stress between T2 and T3, contradicting Hypothesis 2. As the strict restrictions during the initial phase of the pandemic (T1–T2) were eased, some young individuals were expected to be eager to make up for missed social opportunities and activities by extensively engaging with peers and leisure activities, for example. This seems plausible, as adolescence is a phase of individuation and social exploration (e.g., Branje et al., 2012). Relationships outside the family are essential for adolescents’ development, such as identity formation and emotional growth. However, pandemic restrictions limited these interactions more than what seems typically necessary for young people (Kozák et al., 2023; Parent et al. 2021). Further, some research findings prior to the pandemic have provided indications of the negative effects of organized free-time activities, showing that leisure activities, especially when the intensity and involvement become too high, can have negative consequences for problematic peer interactions and feelings of depression (Matjasko et al., 2019; Randall & Bohnert, 2012). In the context of the post-acute phase of the pandemic, these findings, suggest that the (potentially excessive) involvement of adolescents in social interactions - although generally viewed as positive - may also be linked to increased stress.

### The Moderating Role of Gender

Concerning Hypothesis 3, the findings show gender-based differences in the levels of perceived stress and in both dimensions of depressiveness. However, there are only slight differences in how perceived stress and depressiveness relate to each other over time. Women reported higher average levels of perceived stress and negative affect, with more pronounced increases in these dimensions, but lower levels and a less marked increase in positive affect. These findings are consistent with those of previous research on variations in the development of depressive symptoms, with women exhibiting greater vulnerability than men (Hafstad et al., 2022). Biological and hormonal differences, along with differences in coping strategies, stress perception, and methods of maintaining interpersonal relationships, may explain these gender differences (Kuehner, 2017). Further, the vulnerability of male adolescents might have manifested in other dimensions, such as social behavior (Thakur et al., 2023) or how they communicated mental health problems (Rnic et al., 2023).

However, the longitudinal relationship between perceived stress and depressiveness revealed unexpected findings: For male but not for female adolescents, those who reported higher levels of negative and/or positive affect at T2 exhibited a more pronounced increase in perceived stress between T2 and T3. Although all adolescents may have encountered similar disruptions during the pandemic, these experiences may have manifested as different processes for perceived stress and mental health issues in male and female adolescents. Given the unique elements of COVID-19, it is challenging to contextualize this gender-specific pattern within the existing body of knowledge especially as no study has yet examined the stress-depression link with gender as a moderating factor. A post-hoc explanation may relate to gender-specific (habitual) behaviors and available resources during stressful circumstances (Martinez-Hernaez et al., 2016). Here, women might have been better able to manage and communicate their stress experiences and might have been more likely to ask and receive help from others sooner than men or might have been more successful in engaging in and maintaining social connections. Men, on the other hand, might stick with unsuccessful coping strategies (such as staying busy, and avoidant, distracting, and numbing strategies) that can increase psychological stress and, in the long term, be detrimental to health and well-being (see also, Graves et al., 2021).

### Limitations

Although this research has numerous merits, it has several limitations. *First*, perceived stress and the two subdimensions of depressiveness were assessed using concise self-report evaluations, allowing only selective insights into these constructs. Thus, expanding the number of items and facets of individuals’ emotional states and perceived stress would provide deeper insight, especially as lockdowns may have influenced not only positive or negative affect but also other dimensions of emotional well-being (Gniewosz, 2023). However, the limited number of items was necessary due to the reliance on online assessments during COVID-19, when in-person data collection was not feasible due to contact restrictions. Further, a more concise measurement instrument was expected to help maintain participant motivation and keep the burden for participation as low as possible (see Walper & Reim, 2020). *Second*, only adolescents’ subjective responses were recorded, which could have been subject to bias when compared to alternative sources of information. Employing multiple informants for ratings might offer more dependable measurements, but this approach is typically unfeasible in large longitudinal survey studies due to economic constraints. *Third*, the present study is based on information from young adults who voluntarily participated in an additional COVID-19 web survey during a time of high stress. Although extensive efforts were made during the implementation of the COVID-19 web survey to reach a broad and diverse sample (financial compensation, short and time-saving questionnaire; see Walper et al., 2020), it is important to note that the survey may not capture the perspectives of those who were unable to participate in the online survey due to extreme pressure and stress during the pandemic. Nevertheless, the data collected showed no specific dependencies on missing values, as indicated by the non-significant MCAR test. Further the findings are of great value as it provides valuable insights into the pandemic’s challenges and experiences on this specific age group. *Fourth*, our sample comprised a broad age range (15–18 years), raising the possibility of age-specific differences. Nevertheless, all participants belonged to the same birth cohort (born 2001–2003). Further, in all models and the general changes specified therein, age was controlled for over time, making it unlikely that these results were solely due to normative developmental processes (Hawes et al., 2022). *Finally*, more complex models are conceivable, by enhancing model specifications and incorporating additional variables. For instance, for a clearer insight into how perceived stress and depressiveness interact dynamically over time, it is valuable to additionally focus on the relation between the change variables themselves (e.g., LCSM-CC; Orth et al. 2021). Stress and depression are interconnected (e.g., Santee et al., 2023), with the assumption that changes in perceived stress can lead to changes in depressive symptoms. Further, more intricate pathways of influence are plausible where, for instance, the links between perceived stress and depressiveness are either mediated or moderated by factors like personality traits, coping strategies, parental support, or other variables. For instance, research has shown that positive and supportive relationships with parents were associated with reduced perceived stress and improved adjustment during the pandemic (Samji et al., 2022).

## Conclusion

COVID-19 brought significant mental health challenges for adolescents and young adults. While studies have acknowledged the impact of pandemic-related stress on depressiveness, our understanding remains limited due to a lack of long-term investigations into the interplay between perceived stress and depressiveness throughout the pandemic. These relations, including gender-specific differences, were examined using general and multigroup LCSMs. The results underscore the substantial impact of both pre-existing and pandemic-related stress on adolescents’ feelings of depression and how stress increases their susceptibility to depressiveness. Notably, these relationships are not strictly unidirectional, as positive affect also affected perceived stress during the pandemic. Further, the findings suggest gender-specific patterns: While women seemed to have been more vulnerable to perceived stress and depressiveness during the pandemic, the processes underlying the interconnected relationship between stress and depressiveness were more pronounced for men. This emphasizes a gender-specific perspective concerning COVID-19’s effects on adolescents’ mental health. From a research perspective, there is an urgent need to further explore the gender-specific developmental trajectories of young people following COVID-19. Practically speaking, the results point to the need for tailored strategies and interventions that support young people during challenging times.

### Pre-registration

The study was preregistered in the Open Science Framework (OSF) on October 28th, 2023 and comprised research questions and hypotheses, data description, planned analyses and variables as well as a description of prior knowledge of the data. The R-code for the tests and models (i.e., tests of longitudinal measurement invariance, LCS models) as well as additional material (i.e., supplemental material, item list) is also available in the Open Science Framework (https://osf.io/mj924/).

## Supplementary information

Supplementary Information

## Data Availability

The datasets generated and analyzed during the current study are available in the pairfam repository at http://www.pairfam.de/en/data/data-access/.
