# Supplementary material for: Clarifying the Links between Perceived Stress and Depressiveness: a Longitudinal Study of COVID-19’s Effects on Adolescents in Germany
Source: J Youth Adolesc. 2024 May 24;53(10):1–15. doi: 10.1007/s10964-024-02012-8 (PMC11413075; doi:10.1007/s10964-024-02012-8)
Supplement: Supplementary file 1 — Supplementary Information [file 10964_2024_2012_MOESM1_ESM.docx]

# Supplementary Figures & Tables

**“Clarifying the Links Between Perceived Stress and Depressiveness: A Longitudinal Study of COVID-19’s Effects on Adolescents in Germany”**

**Figure S1**

*Overview of Covid-19 waves and measurement points*

**
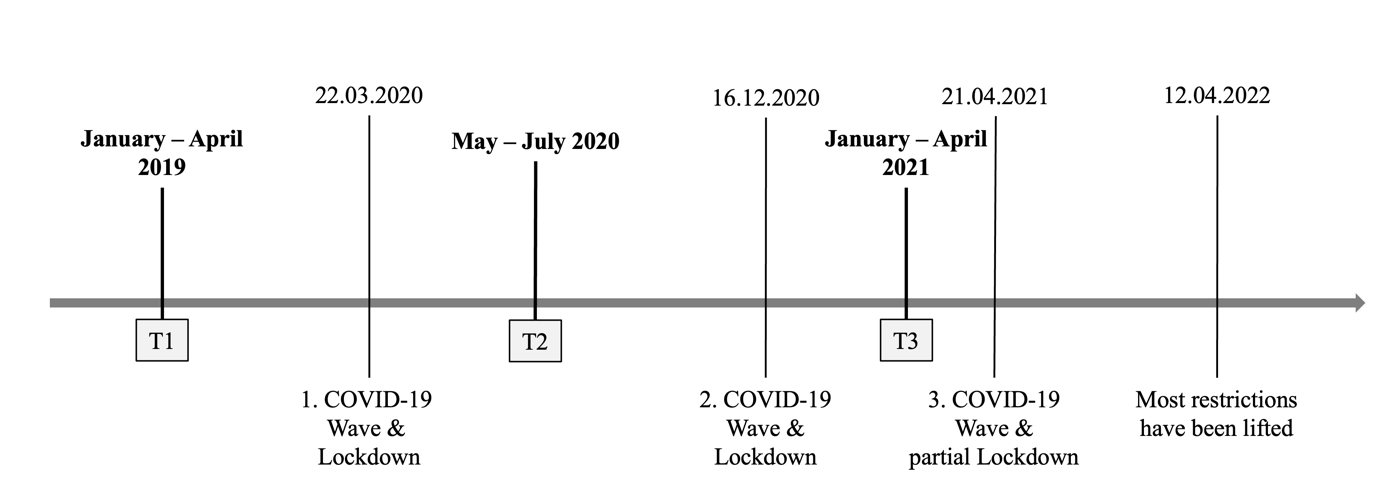
**

**Table S1.** *Exact Item and Scale Information*

| **Variable/Scale** | **Nb. Item** | **Item Label** | **Value** |
| --- | --- | --- | --- |
| Negative affect  (T1 to T3) | Item 1 (cor47i1 / per2i1) | My mood was melancholic | 1 = *almost never* to 4 = *almost always* |
|  | Item 2 (cor47i3 / per2i3) | I felt depressed | 1 = *almost never* to 4 = *almost always* |
|  | Item 3 (cor47i6 / per2i6) | My mood was gloomy | 1 = *almost never* to 4 = *almost always* |
| Positive affect  (T1 to T3) | Item 1 (cor47i2 / per2i2) | I felt happy | 1 = *almost never* to 4 = *almost always* |
|  | Item 2 (cor47i7 / per2i7) | I felt good | 1 = *almost never* to 4 = *almost always* |
|  | Item 3 (cor47i8 / per2i8) | I felt secure | 1 = *almost never* to 4 = *almost always* |
| Perceived stress  (T1 to T3) | Item 1 (cor11i5 / per4i5) | How did you feel in the last four weeks?: Stressed | 1 = *not at all* to 5 = *absolutely* |
|  | Item 2 (cor11i6 / per4i6) | How did you feel in the last four weeks?: Overburdened | 1 = *not at all* to 5 = *absolutely* |
|  | Item 3 (cor11i7 / per4i7) | How did you feel in the last four weeks?: Under pressure | 1 = *not at all* to 5 = *absolutely* |
| Gender (T1) | sex | Reported gender | 1 = *male* to 2 = *female* |
| Age (T1) | age | Age in years | 15 = *15 years* to 18 = *18 years* |
| School track (T1) | GymA11 | School Track | 1 = *low/middle* (*Haupt-/Realschule*) vs. 2 = *high* (*Gymnasium*) |
| Education of family (T1) | FamEduc | Highest educational background of family at T1 | 1 = *low/middle* vs. 2 = *high* |

*Note*. Information originated from the pairfam scales manual (Brüderl et al., 2020; Walper et al., 2020)

**Table S2a**

*Longitudinal Measurement Invariance of Negative Affect*

|  | **Fit Index** | | | | | | | **Difference** | |
| --- | --- | --- | --- | --- | --- | --- | --- | --- | --- |
|  | **𝜒^2^** | ***df*** | ***p*** | **SRMR** | **TLI** | **CFI** | **RMSEA** | **ΔCFI** | **ΔRMSEA** |
| **Negative Affect (across T1, T2, and T3)** | | | | | | | | | |
| Configural | 20.204 | 15 | 0.151 | 0.021 | 0.992 | 0.997 | 0.026 |  |  |
| Weak | 23.542 | 19 | 0.210 | 0.024 | 0.995 | 0.997 | 0.022 | < .001 | .004 |
| Strong | 26.941 | 23 | 0.252 | 0.025 | 0.996 | 0.998 | 0.019 | -.001 | .003 |
| **Strict^** | **33.001** | **29** | **0.274** | **0.025** | **0.997** | 0.997 | **0.017** | **< .001** | **.002** |

*Note.* *N* = 673. Robust ML estimator was used, with FIML for handling missing data. CFI = Comparative fit index; TLI = Tucker–Lewis index; RMSEA = Root mean squared error of approximation; SRMR= Standardized root mean squared residual. Model comparison was as follows: configural vs. weak model, weak vs. strong model, strong vs. strict model. ^Invariance Model (in bold) represents the best fitting model (= level of invariance). According to recommended guidelines, a ∆CFI ≤ 0.01 and ∆RMSEA ≤ .015 indicate that the stricter invariance model should be retained (Putnick & Bornstein, 2016).

**Table S2b**

*Longitudinal Measurement Invariance of Positive Affect*

|  | **Fit Index** | | | | | | | **Difference** | |
| --- | --- | --- | --- | --- | --- | --- | --- | --- | --- |
|  | **𝜒^2^** | ***df*** | ***p*** | **SRMR** | **TLI** | **CFI** | **RMSEA** | **Δ CFI** | **Δ RMSEA** |
| **Positive Affect (across T1, T2, and T3)** | | | | | | | | | |
| Configural | 29.559 | 15 | 0.021 | 0.020 | 0.980 | 0.991 | 0.042 |  |  |
| Weak | 33.871 | 19 | 0.027 | 0.024 | 0.983 | 0.991 | 0.038 | < .001 | .004 |
| Strong | 36.115 | 21 | 0.029 | 0.026 | 0.985 | 0.991 | 0.036 | < .001 | .002 |
| **Strict^** | **39.269** | **27** | **0.099** | **0.026** | **0.990** | **0.993** | **0.029** | **-.002** | **.007** |

*Note.* *N* = 673. Robust ML estimator was used, with FIML for handling missing data. CFI = Comparative fit index; TLI = Tucker–Lewis index; RMSEA = Root mean squared error of approximation; SRMR = Standardized root mean squared residual. Model comparison was as follows: configural vs. weak model, weak vs. strong model, strong vs. strict model. ^Invariance Model (in bold) represents the best fitting model (= level of invariance). One item (per2i2/cor47i2) was not equated over time. According to recommended guidelines, a ∆CFI ≤ 0.01 and ∆RMSEA ≤ .015 indicate that the stricter invariance model should be retained (Putnick & Bornstein, 2016).

**Table S2c**

*Longitudinal Measurement Invariance of Perceived Stress*

|  | **Fit Index** | | | | | | | **Difference** | |
| --- | --- | --- | --- | --- | --- | --- | --- | --- | --- |
|  | **𝜒^2^** | ***df*** | ***p*** | **SRMR** | **TLI** | **CFI** | **RMSEA** | **ΔCFI** | **ΔRMSEA** |
| **Perceived Stress (across T1, T2, and T3)** | | | | | | | | | |
| Configural | 16.14 | 15 | 0.427 | 0.014 | 0.99 | 0.98 | 0.012 |  |  |
| Weak | 19.01 | 19 | 0.484 | 0.016 | 0.99 | 0.98 | 0.001 | < .001 | .011 |
| Strong | 21.77 | 21 | 0.438 | 0.017 | 0.98 | 0.99 | 0.008 | -0.01 | -.007 |
| **Strict^** | **35.47** | **27** | **0.201** | **0.021** | **0.96** | **0.98** | **0.023** | **.01** | **-.015** |

*Note.* *N* = 673. Robust ML estimator was used, with FIML for handling missing data. CFI = Comparative fit index; TLI = Tucker–Lewis index; RMSEA = Root mean squared error of approximation; SRMR= Standardized root mean squared residual. Model comparison was as follows: configural vs. weak model, weak vs. strong model, strong vs. strict model. ^Invariance Model (in bold) represents the best fitting model (= level of invariance). One item (per4i5/cor11i5) was not equated over time. According to recommended guidelines, a ∆CFI ≤ 0.01 and ∆RMSEA ≤ .015 indicate that the stricter invariance model should be retained (Putnick & Bornstein, 2016).

**Table S3a**

*Group-Based Measurement Invariance of Negative Affect*

|  | **Fit Index** | | | | | | | **Difference** | |
| --- | --- | --- | --- | --- | --- | --- | --- | --- | --- |
|  | **𝜒^2^** | ***df*** | ***p*** | **SRMR** | **TLI** | **CFI** | **RMSEA** | **ΔCFI** | **ΔRMSEA** |
| **Negative Affect (across T1, T2, and T3)** | | | | | | | | | |
| Configural | 35.312 | 30 | 0.231 | 0.026 | 0.991 | 0.996 | 0.027 |  |  |
| Weak | 45.155 | 40 | 0.265 | 0.035 | 0.994 | 0.997 | 0.023 | -.001 | .004 |
| Strong | 81.951 | 50 | 0.003 | 0.044 | 0.969 | 0.987 | 0.038 | .01 | -.015 |
| **Strict^** | **99.701** | **65** | **0.004** | **0.047** | **0.974** | **0.977** | **0.046** | **.01** | **-.008** |

*Note.* *N* = 673. Invariance model was based on longitudinal invariant model (see Table S3a). Robust ML estimator was used, with FIML for handling missing data. CFI = Comparative fit index; TLI = Tucker–Lewis index; RMSEA = Root mean squared error of approximation; SRMR= Standardized root mean squared residual. Model comparison was as follows: configural vs. weak model, weak vs. strong model, strong vs. strict model. ^Invariance Model (in bold) represents the best fitting model (= level of invariance). According to recommended guidelines, a ∆CFI ≤ 0.01 and ∆RMSEA ≤ .015 indicate that the stricter invariance model should be retained (Putnick & Bornstein, 2016).

**Table S3b**

*Group-Based Measurement Invariance of Positive Affect*

|  | **Fit Index** | | | | | | | **Difference** | |
| --- | --- | --- | --- | --- | --- | --- | --- | --- | --- |
|  | **𝜒^2^** | ***df*** | ***p*** | **SRMR** | **TLI** | **CFI** | **RMSEA** | **ΔCFI** | **ΔRMSEA** |
| **Positive Affect (across T1, T2, and T3)** | | | | | | | | | |
| Configural | 48.043 | 30 | 0.02 | 0.032 | 0.971 | 0.988 | 0.047 |  |  |
| Weak | 57.692 | 40 | 0.035 | 0.041 | 0.979 | 0.988 | 0.04 | < .001 | .007 |
| Strong | 81.682 | 48 | 0.002 | 0.049 | 0.966 | 0.977 | 0.05 | .01 | -.01 |
| **Strict^** | **104.27** | **63** | **0.001** | **0.066** | **0.968** | **0.972** | **0.049** | **.005** | **.001** |

*Note.* *N* = 673. Invariance model was based on longitudinal invariant model (see Table S3b). Robust ML estimator was used, with FIML for handling missing data. CFI = Comparative fit index; TLI = Tucker–Lewis index; RMSEA = Root mean squared error of approximation; SRMR= Standardized root mean squared residual. Model comparison was as follows: configural vs. weak model, weak vs. strong model, strong vs. strict model. ^Invariance Model (in bold) represents the best fitting model (= level of invariance). One item (per2i2/cor47i2) was not equated over time. According to recommended guidelines, a ∆CFI ≤ 0.01 and ∆RMSEA ≤ .015 indicate that the stricter invariance model should be retained (Putnick & Bornstein, 2016).

**Table S3c**

*Group-Based Measurement Invariance of Perceived Stress*

|  | **Fit Index** | | | | | | | **Difference** | |
| --- | --- | --- | --- | --- | --- | --- | --- | --- | --- |
|  | **𝜒^2^** | ***df*** | ***p*** | **SRMR** | **TLI** | **CFI** | **RMSEA** | **ΔCFI** | **ΔRMSEA** |
| **Perceived Overload (across T1, T2, and T3)** | | | | | | | | | |
| Configural | 28.972 | 30 | 0.519 | 0.02 | 0.999 | 0.999 | 0.020 |  |  |
| Weak | 47.664 | 40 | 0.189 | 0.033 | 0.993 | 0.996 | 0.026 | .003 | -.006 |
| Strong | 56.886 | 48 | 0.178 | 0.035 | 0.994 | 0.996 | 0.026 | < .001 | < .001 |
| **Strict^** | **70.895** | **63** | **0.231** | **0.035** | **0.996** | **0.995** | **0.021** | **.001** | **.005** |

*Note.* *N* = 673. Invariance model was based on longitudinal invariant model (see Table S3c). Robust ML estimator was used, with FIML for handling missing data. CFI = Comparative fit index; TLI = Tucker–Lewis index; RMSEA = Root mean squared error of approximation; SRMR= Standardized root mean squared residual. Model comparison was as follows: configural vs. weak model, weak vs. strong model, strong vs. strict model. ^Invariance Model (in bold) represents the best fitting model (= level of invariance). One item (per4i5/cor11i5) was not equated over time. According to recommended guidelines, a ∆CFI ≤ 0.01 and ∆RMSEA ≤ .015 indicate that the stricter invariance model should be retained (Putnick & Bornstein, 2016).

**Table S4**

*Overview of the Specification of the Bivariate LCSM*

| **Step** | **Model** | **Aim** | **Specified Model** | **Detailed Specification** | **Model Fit** |
| --- | --- | --- | --- | --- | --- |
| 1 | Univariate LCSM  Based on overall sample | To estimate “pure” (proportional and constant) change without exogenous variables or coupling parameters | Model 1 (BM_PS_1 & Multigroup Model):  Perceived stress (PS) | Latent changes for PS (= perceived stress), NA (= negative affect), and PA (= positive affect) could be differentiated into constant and proportional effects (Kievit et al., 2018), whereby the constant effects (i.e., slope of perceived stress = SPs) were fixed parameters that represented global changes across all time points as a measure of overall change.  Proportional changes (i.e., change in PS between T1 and T2 = ΔPS_12_), which were more “local” changes in the variables (McHugh Power et al., 2019), represented the changes relative to the previous states of the variables (Kievit et al., 2018). These latent change scores represented the change in a variable between two time points (i.e., ΔPS_12_) by first measuring the variable at T2 (i.e., PS at T2 = PS_T2_) with a factor loading fixed at 1 and then introducing a beta or feedback parameter (i.e., β_p_). This allowed us to measure the impact of the level of the variable at T1 on the level at T2 (Kievit et al., 2018). Notably, the beta parameters were equally estimated over time.  The latent change scores of ΔPS, ΔNA, and ΔPA were based on latent indicator variables (PS_T1_, PS_T2_, PS_T3_, NA_T1_, NA_T2_, NA_T3_, PA_T1_, PA_T2_, PA_T3_) and were specified from scores at T1 (baseline), with changes modeled between T1 and T2 (before COVID-19), between T2 and T3 (pre-COVID-19 to first wave of COVID-19), and between T3 and T4 (first to second measurement point during COVID-19).  To identify gender-specific values in the change scores, a multigroup model for each univariate LCSM was specified. Gender served as the moderator. For each univariate multigroup model, the measurement model was specified as invariant across groups. The means and variances of latent changes were freely estimated across groups. | BM_PS_1: *χ^2^*(*2*) = 1.740, *p* = .410, RMSEA = .010, CFI = .998, TLI = .990  Multigroup univariate LCSM  MG_univariate_BM_PS_1: *χ^2^*(*6*) 4.42, *p* = .621, RMSEA = .010, CFI =.998, TLI = .995 |
|  |  |  | Model 2 (BM_NA_2 & Multigroup Model):  Negative affect (NA) |  | BM_NA_2: *χ^2^*(*2*) = 4.03, *p* = .133, RMSEA = .039, CFI= .993, TLI = .980  Multigroup univariate LCSM  MG_univariate_BM_NA_2: *χ^2^*(*6*) 9.763, *p* = .135, RMSEA = .043, CFI = .987, TLI = .973 |
|  |  |  | Model 3 (BM_PA_3 & Multigroup Model):  Positive affect (PA) |  | BM_PA_3:*χ^2^*(*2*) = 4.17, *p* = .133, RMSEA = .040, CFI = .993, TLI = .979  Multigroup univariate LCSM  MG_univariate_BM_PA_3: *χ^2^*(*6*) 15.64, *p* = .016, RMSEA = .069, CFI = .968, TLI = .935 |
| 2 | Bivariate latent change score models  Based on overall sample | To specify the bidirectional paths between two variables across time  To obtain a parallel estimation of (proportional and constant) change in two variables  To add coupling parameters between variables  To add three exogenous variables (age, school, family education) | Model 1 (M_PSNA_1):  PS and NA | The bivariate LCSMs were based on the univariate LCSMs, combining the latent changes of two variables (PA and PS, NA and PS).  In a bivariate dual LCSM, a coupling parameter was specified to represent the time-dependent effects of one variable on the latent change of the other (McArdle, 2009). As a result, the change (i.e., ΔPS_12_) in this model was a function of the constant (i.e., the slope of PS across the four time points = SPs), the prior value of the variable (i.e., PS at T1 = PS_T1_), and the prior value of the coupled variable (i.e., NA at T1 = NA_T1_).  Further, three exogenous variables were added to test the predictive value of adolescents’ age, school track, and education of the family on proportional changes in NA, PA, and PS (ΔPS_12_ at T1 to T2, ΔPS_23_ at T2 to T3; ΔNA_12_ at T1 to T2, ΔNA_23_ at T2 to T3; ΔPA_12_ at T1 to T2, ΔPA_23_ at T2 to T3). | M_PSNA_1: *χ^2^* (*7*) = 8.754, *p* = .272, RMSEA = .019, SRMR = .016, CFI = .998, TLI = .990 |
|  |  |  | Model 2 (M_PSPA_2):  PS and PA |  | M_PSPA_2: *χ^2^* (*7*) = 21.776, *p* = .059, RMSEA = .026, SRMR = .016, CFI = .988, TLI = .964 |

**Table S5**

*Overview of the Specification of the Bivariate LCSM (Continuation of Table S5)*

| **Step** | **Model** | **Aim** | **Specified Model** | **Detailed Specification** | **Model Fit** |
| --- | --- | --- | --- | --- | --- |
| 3 | Multigroup bivariate latent change score models  Subsamples of female vs. male adolescents | To base the model on bivariate latent change score models (with coupling parameters and exogenous variables)  To add gender as a moderator  To obtain a parallel estimation of the bivariate LCSMs across gender groups | Model 1 (MG_PSNA_1):  PS and NA across gender groups | The multigroup model was based on the bivariate LCSM specified in Step 2.  The LCSM was estimated simultaneously in both groups and included all coupling parameters and exogenous variables.  The measurement models of the change scores (ΔPS_12_ at T1 to T2, ΔPS_23_ at T2 to T3; ΔNA_12_ at T1 to T2, ΔNA_23_ at T2 to T3; ΔPA_12_ at T1 to T2, ΔPA_23_ at T2 to T3) were set to be invariant across the gender groups.  The structural model, including the coupling parameters and parameters of the exogenous variable, were freely estimated across both groups.  Gender-specific differences were tested using the Wald test. | MG_PSNA_1: *χ^2^* (*14*) = 22.87, *p* = .021, RMSEA = .043, SRMR = .027, CFI = .988, TLI = .945 |
|  |  |  | Model 2 (MG_PSPA_2):  PS and PA across gender groups |  | MG_PSPA_2: *χ^2^* (*14*) = 39.47, *p* = .021, RMSEA = .039, SRMR = .034, CFI = .980, TLI = .941 |
